# Community Pharmacies in Poland—The Journey from a Deregulated to a Strictly Regulated Market

**DOI:** 10.3390/ijerph17238751

**Published:** 2020-11-25

**Authors:** Marcin Wiśniewski, Urszula Religioni, Piotr Merks

**Affiliations:** 1 Association of Pharmacists of Employers of Polish Pharmacies, 01-711 Warsaw, Poland; kontakt@aptekarze.org.pl; 2 Collegium of Business Administration, Warsaw School of Economics, 02-513 Warsaw, Poland; urszula.religioni@gmail.com; 3 Cardinal Stefan Wyszyński University in Warsaw Faculty of Medicine, Collegium Medicum, 01-938 Warsaw, Poland; 4 Department of Pharmaceutical Technology, Faculty of Pharmacy, Collegium Medicum in Bydgoszcz, 85-067 Bydgoszcz, Poland; 5 Employed Pharmacist in Europe (EPhEU) Verband Angestellter Apotheker Österreichs (VAAÖ) Berufliche Interessenvertretung Spitalgasse 31/4, Vienna 1090, Austria

**Keywords:** community pharmacy, pharmacy market, pharmacist, pharmacy regulations

## Abstract

Community pharmacies are the primary entities providing drugs to individual patients in Poland. The pharmacy market has been changing for many years due to significant changes in market regulations. These changes significantly affect the profitability of pharmacies, which may impact the quality of pharmacotherapy. The small number of pharmacies, which resulted from changes in the law in 2017, can influence the level of patient care. The article presents the community pharmacies market in Poland. Particular attention is paid to the legal regulations affecting community pharmacies and the impact of these regulations on the overall shape of the market. The Polish system’s specificity, including the pharmacy market indicators, has been compared with data from other European Union countries.

## 1. Introduction

Drug policy is an integral part of health policy being a combination of medical and economic areas. Providing patients with effective and safe therapy seems to be one of the essential drug policy elements. Thus, appropriate drug policy is one of the most important aspects of public health [1]. In the area of drug policy, actions of governments of all countries should be directed at improving the population health and achieving better health outcomes [2], which is impossible without a rational drug policy.

In that context, the enormous role of a community pharmacist in the healthcare system should be emphasised. The pharmacist is a patient’s advisor in the field of medications. Patients’ access to pharmaceutical care is a guarantee of drug safety and achieving the expected effects of therapy [3,4]. The role of the pharmacist seems to be particularly important in the time of the COVID-19 pandemic. Many emergency cases have resulted in a significant inefficiency of health care systems, which is also visible in Poland. Importantly, in April 2020, legal changes allowed pharmacists to prescribe the reimbursed fully paid drug pro auctore and pro familiae and fully paid drug for patients in health emergency up to a maximum of 180 days of therapy. This change was of particular importance during the pandemic when patients had significant difficulties in accessing doctors. Before that, pharmacists could prescribe a drug only in the situation of a sudden threat to the patient’s health or life, and the patient could only receive the smallest possible packaging of the drug.

In the Polish health care system, the central place of drug dispensing to patients is the community pharmacy. However, some products can also be purchased at limited-service pharmacies or non-pharmacy outlets. On the Polish community pharmacy market, a significant increase in the number of pharmacies and pharmacy chains has been observed for many years [5]. This situation was a stimulus for changes in legal regulations. In 2012, the so-called “Pharmacy for the Pharmacist” Act was introduced aimed at tightening regulation of the pharmaceutical market [6].

This article characterises the market of community pharmacies in Poland and the possible consequences of the market regulations for public health. The article presents the situation of the community pharmacy market before and after the introduction of the “Pharmacy for the Pharmacist” Act. The impact of legal changes for pharmacy owners in Poland was highlighted, referring to demographic and economic measures.

## 2. Community Pharmacies Market Regulations in Poland

Community pharmacies play a crucial role in the drug distribution system in Poland. The primary legal acts regulating the functioning of community pharmacies in Poland are the Pharmaceutical Law Act [7] and the Reimbursement Act [8]. Many detailed regulations also restrict the market.

The Pharmaceutical Law Act [7] indicates that the pharmacy is a public health protection institution in which authorized persons provide pharmaceutical services, such as dispensing medicinal products and medical devices, preparing prescription drugs, providing information on medicinal products and medical devices. Currently, running a pharmacy is a regulated activity. The necessary condition for running a pharmacy in Poland is obtaining an appropriate permit. The State Provincial Pharmaceutical Inspector issues permissions to operate pharmacies.

Retail trade in medicinal products is also carried out in limited-service pharmacies and non-pharmacy outlets, including grocery stores. Non-pharmacy outlets may only offer OTC drugs. It should be emphasized that trade in drugs in this segment is subject to few regulations or controls. In turn, community pharmacies are controlled by 16 Provincial Pharmaceutical Inspectors subordinate to the provincial governors and, substantially, to the Main Pharmaceutical Inspector. The lack of a single supervisor leads to a dilution of responsibility for the state of the market. Therefore, an important guideline for the future is to improve the inspection by verticalisation. Pharmaceutical Inspectors’ tasks include control of pharmacy activities, quality control of prescription drugs, issuing permits to operate pharmacies, monitoring deficiencies, and reasons for unavailability of retail drugs. Audits carried out by the Supreme Chamber of Control in Poland indicate, however, that the current number of inspectors is not sufficient for the adequate performance of their mandated activities [9].

Pharmaceutical law adopted in 2001 [7] stated that the concession to operate a community pharmacy may be obtained only by a pharmacist and that one pharmacist could have only one concession. A year later, the regulations were liberalized, allowing any person or company to open a pharmacy. It was possible to run many pharmacies, but a maximum of 10% of pharmacies in Poland. This situation has led to the formation of many so-called chain pharmacies that were not owned by pharmacists. Ten percent limit ownership was the first anti-concentration limit in the Polish pharmaceutical law. In 2004, with Poland’s accession to the European Union, the anti-concentration limit was changed from 10% pharmacies in the country up to 1% pharmacies in the province.

In 2017, an amendment to the Pharmaceutical Law (the so-called “Pharmacy for the Pharmacist”) was adopted. This act allows new pharmacies to be opened only by pharmacists, limits the number of pharmacies, and sets geographical and demographic limits. Thus, currently, it is prohibited in Poland to create a pharmacy chain. One pharmacist (or a partnership of pharmacists) may run a maximum of four pharmacies. The pharmacy chains existing until 25 June 2017 can still operate in the market, but they cannot expand [10]. Existing pharmacies can only be resold to pharmacists or pharmacists’ partnerships.

The drug reimbursement system, together with the definition of rigid margins and prices, is also strictly regulated in Poland [11]. According to the law, drugs with no over-the-counter (OTC) equivalent or drugs having an over-the-counter equivalent which must be used for 30 days, may be reimbursed. Prices of reimbursed drugs are the same throughout the country; the Reimbursement Act introduced fixed prices and fixed margins in the pharmacy channel. The reimbursement decision determines the official price and wholesale margins. Prices for full-price drugs, OTC drugs and the rest of the pharmacy assortment remain unregulated.

In Europe, there are two legal models for pharmacy markets: strictly regulated (laws regarding the establishment of pharmacies, ownership, distribution, density, etc.) and deregulated—with a small degree of market regulation.

In most European countries, as in Poland, the pharmaceutical market is heavily regulated [12], and the pharmacy can only be run by a pharmacist (this applies to over 70% of pharmacies throughout the EU). Demographic limits set by law or decision of an office in a specific location apply in 19 European countries. There are no demographic limits in eight EU countries, namely Bulgaria, the Czech Republic, Slovakia, the Netherlands, Germany, Sweden, Lithuania, and Ireland [13].

In turn, countries adopting the deregulated model aimed to increase the availability of drugs. In fact, deregulation has led to the opening of new pharmacies, primarily in urban areas. Deregulation of the pharmacy market usually leads to uneven distribution of pharmacies throughout the country, the dominant position of some market participants, the emergence of economic pressure to increase pharmacy turnover by selling over-the-counter and non-drug products [13,14].

The rapid development of the pharmacy chains led to a situation in which pharmacies mainly deal with sales, excluding health promotion. Due to the ban on advertising of pharmacies, promotions, competitions and loyalty programs have been organized to maximize profits in pharmacies. The significant increase in advertising expenditure on OTC drugs and dietary supplements (about 15% per year) is also worrying [15]. These activities could have significantly contributed to the situation where the consumption of OTC drugs and dietary supplements (self-medication) in Poland is one of the highest in the EU per capita. The reason may also be the virtually unlimited availability of these drugs. Almost half of the sales of ibuprofen and paracetamol-containing drugs are in the non-pharmacy channel [13].

The growing number of pharmacies has resulted in a systematic drop in profitability. Profit-oriented entrepreneurs began to look for other sources of income besides their retail margin. This resulted in the creation of an inverted distribution chain that causes problems leading to a lack of availability of many drugs for Polish patients. “Reverse distribution chain” is the sale of drugs in a direction contrary to the supply chain assumptions, i.e., not to patients, but rather to entities that export them abroad for higher profits. Reacting to the illegal export of drugs, manufacturers then limit the amount of supplies or limit them to selected, preferred recipients, which increases the problems with purchasing drugs. Drugs are exported and sold abroad due to the difference in prices between Poland and other European Union countries because drug prices in Poland are among the lowest in Europe [15,16,17]. The announcement of the Minister of Health indicated that the list of drugs at risk of availability in June 2020 included 298 drugs (according to EAN codes).

## 3. The Market of Community Pharmacies in Poland and EU Countries

**Figure 1:**
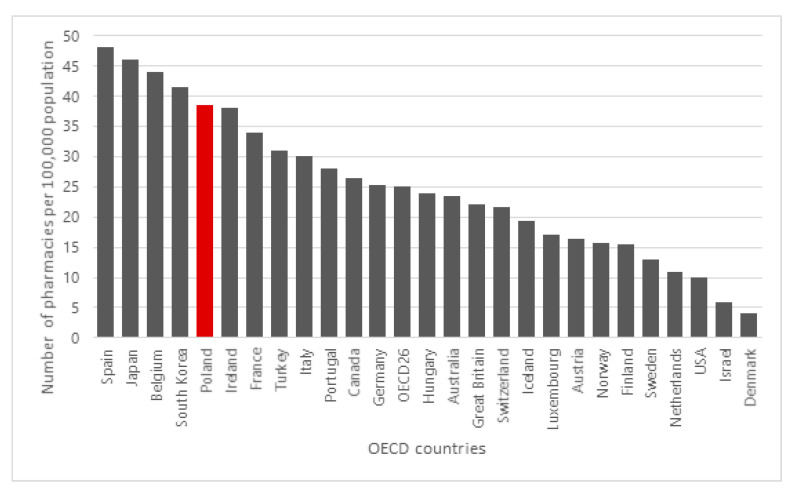
Number of pharmacies per 100,000 population in Poland and other OECD countries.

According to data from the Central Statistical Office, at the end of 2017, there were 13,300 community pharmacies and 1300 thousand pharmacy outlets (thus approximately 14,600 thousand in total) in Poland. After the implementation of the “Pharmacy for the Pharmacist” Act in June 2017, the number of pharmacies increased sharply (due to the implementation of applications for authorization submitted before the act entered), reaching 15,000, and then began to fall to 12,900 thousand at the end of 2018 [13,18]. The number of pharmacies per 100,000 population in Poland is one of the highest among OECD countries (Figure 1).

**Figure 2:**
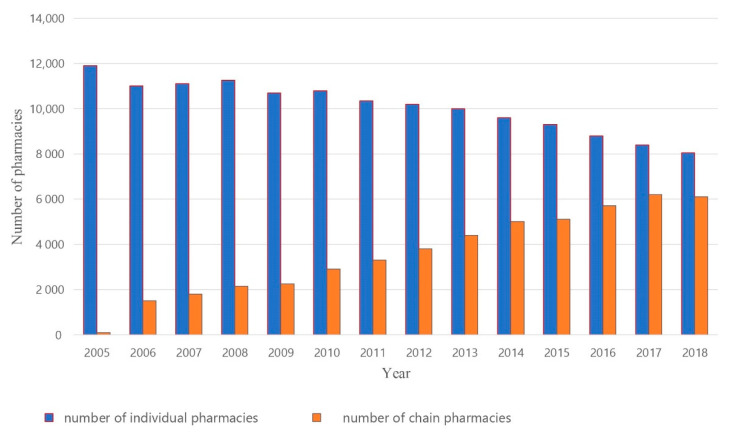
Changes in the number of individual and chain pharmacies in the years 2000–2015.

The pharmacy market is divided between individual and chain pharmacies (defined as five or more pharmacies). There are approximately 8200 individual pharmacies (57% of the market) and 6200 chain pharmacies (43% of the market) [13] (Figure 2).

According to the Central Statistical Office data, in 2017, there was one pharmacy for every 2628 people in Poland. By the end of 2018, this number had increased to 2716 people [18]. This is an incomparably smaller number compared to the European average (4320 inhabitants per pharmacy). In countries with a GDP per capita similar to Poland, the number of people per pharmacy is much higher. For example, in Slovakia, there are approximately 3600 people, in the Czech Republic—approximately 3700 people, in Croatia—approximately 4000 people and in Hungary—approximately 4300 people per pharmacy. In eight other European countries, the number of inhabitants per pharmacy is twice or even three times higher than in Poland [13].

At the end of 2018, 67,100 people worked in community pharmacies, hospital pharmacies, and limited-service pharmacies, including 26,700 pharmacy masters (a decrease of 1.3% per year) and 33,700 pharmaceutical technicians (an increase by 1.0% compared to the previous year) [18].

The profitability of a pharmacy depends on three parameters: the number of patients per pharmacy, the amount of expenditures on drugs per patient, as well as the pharmacy’s margins. The latter is partly regulated (via drug reimbursements) by law. The pharmacy’s income is also affected by other factors, such as the location, factual and friendly staff, epidemiology, etc. The average turnover of a typical pharmacy in Poland is very low compared to other EU countries. This is mainly due to two reasons: the average drug expenditure per inhabitant of Poland is among the lowest in Europe, and the number of inhabitants per pharmacy is relatively small. In 2018, pharmacies achieved a total turnover of PLN 34.5 billion. Individual pharmacies generated a total turnover of approx. PLN 14.2 billion (41.1% of the market value), i.e., on average approximately 144,000 PLN per month per pharmacy. At the same time, chain pharmacies generated a turnover of approximately PLN 20.3 billion (58.9% of the market value), an average of 273,500 PLN per month per pharmacy [13].

Pharmacy margins in Poland are at the average European level, although the low margins on reimbursed drugs mean that trading in this group of drugs is at a loss. Pharmacy margins in Europe range from 14% (France, Luxembourg) to 43% (Italy, Cyprus). In Poland, the average pharmacy margin is around 25%, and in relation to individual segments: around 18.5% for reimbursed drugs (Rx—the margin is established officially, fixed for each drug), around 24.5% for fully-paid prescription drugs (Rx 100%) and 29.5% for products sold freehand [13]. Regulating official margins at too low level shifts the margin to full-paid drugs, OTC, and the remaining assortment of good available at the pharmacy.

Consequently, a typical pharmacy in Greece has a lower turnover than in Poland, where the number of pharmacies is very high and in Latvia (a value similar to that in Poland). In other European countries, pharmacies have higher potential. Pharmacy turnover is at least twice as high in eleven countries. Among countries with a GDP per capita similar to that of Poland, pharmacy turnover is higher—twice as high in the Czech Republic and three times as high in Hungary. Considering the purchasing power of Polish residents, the average turnover of a Polish pharmacy is 926,000 USD a year. Meanwhile, a pharmacy in the Czech Republic reported 1.68 million USD a year and in France 1.99 million USD. Pharmacies generate the highest average turnover in Austria, Denmark, and the Netherlands, respectively, 3.88 million, 3.52 million, and 3.47 million USD due to the low density of pharmacies [13].

**Figure 3:**
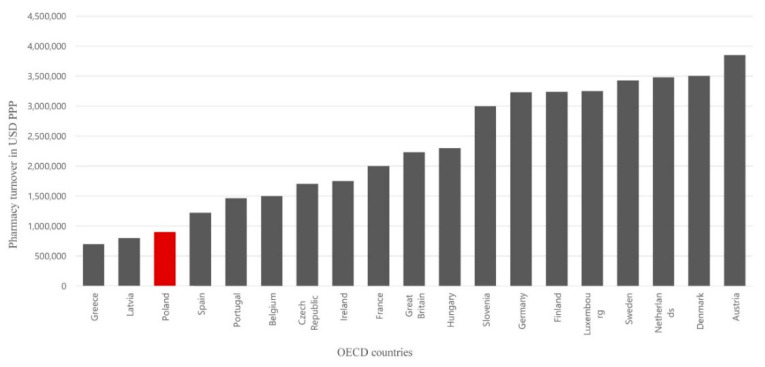
Pharmacy turnover/income in other countries (own analysis based on OECD data, values in USD PPP).

The overall profitability of Polish pharmacies is one of the lowest in Europe (after Greece and Latvia), limiting their ability to perform their public service tasks properly and is the reason for seeking profits outside the margin, including unlawful (e.g., reverse distribution chain) activities. The impoverishment of pharmacies is a significant threat to their independence. It weakens them economically and leads to them passing into the hands of foreign retail chains. A typical pharmacy in Poland generates a turnover twice lower than a pharmacy in the Czech Republic and three times lower than a pharmacy in Hungary (Figure 3). A pharmacy in Slovenia, Germany, Finland, Luxembourg, Sweden, the Netherlands, Austria, and Denmark generates turnover about four times higher than in Poland. 

The average expenditure on drugs (Rx and OTC from the pocket of a patient and payers) per capita in Poland is the lowest among OECD countries and in 2017 was 417 USD, with an average of 564 USD [19]. Only in a few countries is spending on drugs lower than in Poland. The situation may arise from the fact that overall health expenditure in Poland is lower than in other countries and also, because drugs in Poland are among the cheapest in Europe [16].

In 2012, the reimbursement act resulted in an increase in patients’ co-payment for prescription drugs (reimbursed and non-reimbursed) by as much as 5.2 percentage points (from 52.5% to 57.7%), and in the case of reimbursed drugs alone, by 2 percentage points (from 36.7% in 2011 to 38.7% in 2012). In the first quarter of 2013, the co-payment of patients for reimbursed drugs increased to 40.3%. In 2013–2019, the Ministry of Health’s reimbursement policy placed particular emphasis on reducing the prices of drugs covered by pharmacy reimbursement, which translated into price pressure on drug manufacturers related to the frequent publication of reimbursement lists and lowering prices of subsequent generic drugs entering the lists. Market conditions also resulted in a significant drop in the wholesale margin for reimbursed medications during this period. According to PharmaExpert, in 2011 the share of reimbursed drugs accounted for 45% of pharmacy turnover, systematically falling. All these factors ultimately led to a lower level of patient co-payment for reimbursed drugs. Currently, the co-payment of Polish patients for reimbursed drugs is around 27–28%, while the co-payment level for all prescription drugs, according to IQVIA is still very high and amounts to 55.6% [13]. This situation is especially worrying because the World Health Organization (WHO) indicates that exceeding the level of 40% co-payment of patients for drugs means severe restrictions for patients in accessing drugs.

Across OECD countries, funding from governments and compulsory insurance schemes played the largest role in purchasing pharmaceuticals. In 2015, Poles paid the most out-of-pocket for drugs among the OECD countries. According to the OECD, 66% of the costs of the drugs are covered by Polish patients. The situation is similar in Lithuania, and the situation is worse only in Cyprus and Bulgaria. In 2017, funding from governments and compulsory insurance schemes covered 58% of spending on retail pharmaceuticals in Europe. Most of the remainder is financed from out-of-pocket household payments; only 3% of spending is covered by voluntary insurance. In Germany and France, government and compulsory schemes include 80% or more of pharmaceutical costs. By contrast, in Latvia, Poland, and Lithuania, almost two-thirds of pharmaceutical spending was through out-of-pocket payments [13,19].

The costs of running a pharmacy and the low official margin for reimbursed drugs result in a loss of trade in reimbursed drugs. Consequently, pharmacies impose high margins on full-paid drugsand OTC drugs and increase the sale of these products. This situation has a direct impact on the higher turnover of chain pharmacies and is considered as a direct reason for the excessive consumption of pharmaceuticals by Poles. The OECD report indicates very high use of OTC drugs, and high expenses on OTC drugs in Poland, which are almost equal to that on prescription drugs [19].

## 4. The Role of Community Pharmacists in the Healthcare System

In Poland, according to OECD statistics, the number of pharmacists per 100,000 population is 77, while the average of the OECD countries is 83. In Hungary, Slovakia, Estonia, and Sweden, the number of pharmacists is similar to that in Poland. In many countries, the number of pharmacists per 100,000 inhabitants is lower: the Netherlands—21, Denmark—52, and Germany—65. There are also countries with a much higher number of pharmacists: Belgium—124, Italy—117, and Spain—116 [19].

**Figure 4:**
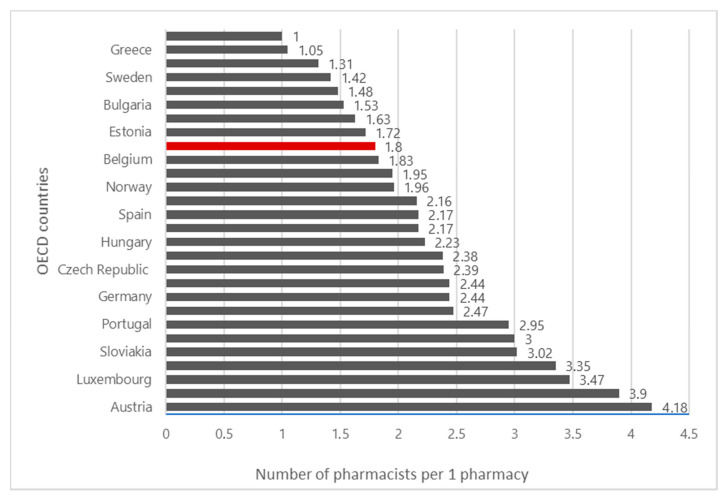
Number of pharmacists per pharmacy in Poland and other OECD countries.

According to the Central Statistical Office, the number of pharmacists per pharmacy in Poland is 1.85. Poland is divided into 16 voivodships (a voivodship is an administrative division unit in Poland). In individual voivodships, the number ranges from 1.43 to 2.22 (data from the Ministry of Health indicate a lower average—1.78 pharmacists per pharmacy). The average for EU countries is 2.40 pharmacists per pharmacy (Figure 4). The particularly alarming situation is in West Pomerania, where there is only one pharmacist per pharmacy. Only two voivodships meet the necessary conditions for staffing pharmacies—Łódź and Pomerania (2.5 pharmacists) [20]. Moreover, over 7500 working pharmacists are approaching or are in retirement age. This represents almost 30% of all the pharmacists working in pharmacies.

It is particularly worrying that, considering that nearly 40% of Poles live in rural areas, only 12.8% of pharmacists and 13% of pharmacy technicians work in these areas. This is due to the lack of geographical and demographic restrictions in the years 2001–2016 when pharmacies were established mainly in cities, which is typical for deregulated markets [13].

The pharmacist’s job aims to protect public health and includes the provision of pharmaceutical care services. Pharmacists are highly qualified specialists in the field of healthcare. Practising the profession of a pharmacist in Poland requires obtaining a master’s degree in pharmacy. Pharmacy studies last 5 years. Additionally, at least 6 months of professional apprenticeship is necessary to practice the profession of pharmacist. Foreign diplomas must be recognized as equivalent to a diploma and a master’s degree in pharmacy from the Republic of Poland. The pharmacist (pharmacist) has a statutory continuous training obligation.

The pharmacists’ knowledge allows them to participate in the treatment process of each patient actively. An important duty of pharmacists is to provide reliable and objective information about drugs. It is estimated that up to 50% of medicinal products are administered incorrectly [21]. It results in a lack of improvement in patients’ health and the occurrence of many side effects of drugs, which contributes to a significant increase in the overall cost of therapy [22]. Pharmacists have extensive knowledge of the physicochemical, pharmacodynamic, and pharmacokinetic properties of drugs. Cooperation of pharmacists with patients and medical staff (pharmaceutical care) is currently considered one of the essential drug management elements, significantly affecting the effectiveness of patient therapy [23,24].

According to the Pharmaceutical Care Network Europe (PCNE), pharmaceutical care is the pharmacist’s contribution to patient individuals’ care to optimize medicines use and improve health outcomes. [25]. In the context of the pharmacist’s work, pharmaceutical care has three main components:

(1) Detection of real or potential drug problems,
(2) Solving real drug problems,
(3) Prevention of drug problems,

and a drug problem is considered as any adverse effect experienced by the patient due to, or probably caused by a drug, that affects or may affect the effects of therapy.

The American Society of Healthcare System Pharmacists (ASHP) indicates that the most common drug problems include: inadequate drug selection, inadequate dosage or the length of use, adverse effects related to the therapy with a particular preparation, drug interactions [26].

In order to optimize pharmacotherapy, it seems necessary to change the approach to the pharmacist’s role from the person dispensing drugs to the person actively participating in the patient’s treatment process and responsible for improving health outcomes [27].

Many studies confirm the effectiveness of pharmacist’s interventions in patients’ therapy, especially for the chronically ill. Pharmaceutical care improves patient’s compliance, expands the patient’s knowledge about the disease and improves diagnostic test results [24,28,29,30,31]. The pharmacist’s help in the proper use of OTC drugs is also of great importance, especially among older, due to the high risk of polyprgamasia (polypharmacy) in this group of patients and the associated health consequences [32].

Pharmaceutical care is underdeveloped in Poland, mainly due to the lack of financing for these services. The remuneration for the pharmaceutical care service is included in the price of the drugs. There are no additional components of the margin. As a consequence, it led to the accumulation of problems related to lack of polypragmasia control, high prevalence of patient self-medication, and non-compliance with medical recommendations [15,33].

It is estimated that about 10–25% of hospital treatment cases are caused by patients not correctly following the recommended pharmacotherapy, and about 30–50% of chronically ill patients do not adequately take their prescribed medications. A significant problem is also observed in the case of incorrect use of painkillers. Serious complications associated with this group of drugs are among the top five most common causes of hospital admissions [34,35].

Undesirable effects of drugs worsen patients’ quality of life, increasing both the number of hospitalizations and deaths. Most of them require treatment, which in turn generates additional costs [35]. Numerous studies show that 30–60% of adverse drug reactions can be prevented. Educating patients on the proper use of drugs is one of the most crucial elements of health education. It is important to personalize the therapy and adapt the treatment to the patient. These activities improve the quality of patient’s life, reduce the side effects of drugs and increase therapy effectiveness [4,36]. Interpersonal communication between a doctor, pharmacist, and the patient is a critical element of pharmacological treatment.

Providing health care requires not only medical but also pharmaceutical knowledge. Thus, the insufficient (very low) number of people employed in pharmacies may become one of the reasons for the decrease in the quality of patient care, including the occurrence of many errors in the field of drug therapy.

Many countries have systematically increased the role of pharmacists in health care. Given the growing costs of health care resulting from the ageing of the population, the extension of life expectancy associated with the increase in the incidence of chronic diseases (e.g., diabetes, COPD) or the shortage of healthcare professionals, the potential of pharmacists is more fully used. Although the main task of pharmacists is to dispense drugs in public pharmacies, they also increasingly provide direct care for patients (e.g., vaccinations, monitoring clinical parameters, support in compliance with medical recommendations [37]. In the years 2000–2015, the number of pharmacists in the OECD nations group increased by 30% [13,19]. The activation of this group increases the number of personnel involved in health care, improve patient’s care, and brings enormous savings.

## 5. Conclusions

Community pharmacies are one of the essential health care facilities in Poland. In recent years, changing legal regulations have resulted in the transformation of the pharmacy market. The effects of these changes were visible to both pharmacy owners and patients. Deregulation of the community pharmacy market in Poland before 2017 rapidly increased the number of pharmacies. This situation significantly reduced turnover, closed many individual pharmacies and increased the number of chain pharmacies. Also, despite the promises to improve Poles’ access to pharmaceutical care services, the regulations have just contributed to the creation of new pharmacies, mainly in cities, which did not meet the assumed goal. Pharmacies began to perform mostly commercial roles, and restrictions on margins caused an increase in the sale of OTC drugs, cosmetics or dietary supplements.

Tighter market regulation, which has already been introduced in 2017, will favour this situation. The introduction of the “Pharmacy for the Pharmacist” Act has significantly reduced the creation of new pharmacies, increasing the prestige of the pharmacist profession. This situation is particularly important taking into account the enormous role of the pharmacist in population health protection, which is particularly noticeable during the COVID-19 pandemic. Detailed legal regulations may result in the more effective functioning of the whole health care system. Further stabilization of the pharmacy market in Poland is expected in the next years.

## References

[1] Csete J., Kamarulzaman A., Kazatchkine M., Altice F.L., Balicki M., Buxton J., Cepeda J., Comfort M., Goosby E., Goulão J. (2016). Public health and international drug policy. Lancet.

[2] Lancaster K. (2014). Social construction and the evidence-based drug policy endeavour. Int. J. Drug Policy.

[3] Gammie T., Vogler S., Babar Z.-U.-D. (2016). Economic Evaluation of Hospital and Community Pharmacy Services. Ann. Pharmacother..

[4] Akinbosoye O.E., Taitel M.S., Grana J., Hill J., Wade R.L. (2016). Improving Medication Adherence and Health Care Outcomes in a Commercial Population through a Community Pharmacy. Popul. Health Manag..

[5] Merks P., Byliniak M., Religioni U., Włodarczak U., Świeczkowski D., Jaguszewski M. Current Situation of the Polish Pharmacy Sector. https://epheu.eu/poland-more-about-pharmacy/.

[6] Zaprutko T., Hromovyk B., Lesyk R., Lesyk L., Kremin Y., Kus K., Kopciuch D., Ratajczak P., Paczkowska A., Nowakowska E. (2020). Pharmacies for the Pharmacists—Ukrainian Fears and Polish Experiences. Sci. Pharm..

[7] Act of 6 September 2001—Pharmaceutical Law. (Journal of Laws 2001 No. 126, Item 1381, As Amended). http://isap.sejm.gov.pl/isap.nsf/download.xsp/WDU20011261381/U/D20011381Lj.pdf.

[8] Act of 12 May 2012 on the Reimbursement of Medicines, Foodstuffs for Particular Nutritional Uses and Medical Devices (Journal of Laws of 2011, No. 122, Item 696, As Amended). http://isap.sejm.gov.pl/isap.nsf/download.xsp/WDU20111220696/U/D20110696Lj.pdf.

[9] The Supreme Chamber of Control, Performing by the State Pharmaceutical Inspection the Tasks Specified in the Pharmaceutical Law. https://www.nik.gov.pl/plik/id,10361,vp,12689.pdf.

[10] World Health Organization (2019). The Legal and Regulatory Framework for Community Pharmacies in the WHO European Region.

[11] Wlodarczyk I., Scahill S.L., Babar Z.U.D. (2017). Pharmaceutical Policy in Poland. Pharmaceutical Policy in Countries with Developing Healthcare Systems.

[12] Vogler S., Habimana K., Arts D. (2014). Does deregulation in community pharmacy impact accessibility of medicines, quality of pharmacy services and costs? Evidence from nine European countries. Health Policy.

[13] Association of Pharmacists of Employers of Polish Pharmacies Pharmacies in Poland—Report. ZAPPA. http://aptekarze.org.pl/wp-content/uploads/2019/05/zappa_raport_2019_19_04_2019_dr.pdf.

[14] Nguyen T.A., Knight R., Roughead E.E., Brooks G., Mant A. (2015). Policy options for pharmaceutical pricing and purchasing: Issues for low- and middle-income countries. Health Policy Plan..

[15] Ministry of Health National Drug Policy for 2018–2022. https://www.gov.pl/attachment/bb36ad25-4342-4000-bfaa-64004c64a62c.

[16] Panteli D., Arickx F., Cleemput I., Dedet G., Eckhardt H., Fogarty E., Gerkens S., Henschke C., Hislop J., Jommi C. (2016). Pharmaceutical regulation in 15 European countries review. Health Syst. Transit..

[17] Main Pharmaceutical Inspectorate Inverted Distribution Chain. https://www.gif.gov.pl/pl/aktualnosci/731,Odwrocony-lancuch-pytania-i-odpowiedzi.html.

[18] Central Statistical Office of Poland Pharmacies and Pharmacy Points in 2018. https://stat.gov.pl/obszary-tematyczne/zdrowie/zdrowie/apteki-i-punkty-apteczne-w-2018-roku,15,3.html.

[19] OECD (2019). Health at Glance 2019: OECD Indicators.

[20] Supreme Pharmaceutical Chamber Report on the Personnel Situation of Pharmacists for Poland. http://www.mpz.mz.gov.pl/wp-content/uploads/sites/4/2018/06/raport_farmaceuci.pdf.

[21] Grześkowiak E. Hospital Pharmacy. https://mz.gov.pl/wwwfiles/ma_struktura/docs/78_farmacja_szpitalna_13072011.pdf.

[22] Gregório J., Russo G., Lapão L.V. (2016). Pharmaceutical services cost analysis using time-driven activity-based costing: A contribution to improve community pharmacies’ management. Res. Soc. Adm. Pharm..

[23] American Pharmacists Association, National Association of Chain Drug Stores Foundation (2008). Medication Therapy Management in Pharmacy Practice.

[24] Abdelhamid E., Awad A., Gismallah A. (2008). Evaluation of a hospital pharmacy-based pharmaceutical care services for asthma patients. Pharm. Pract..

[25] Allemann S.S., Van Mil J.W.F., Botermann L., Berger K., Griese N., Hersberger K.E. (2014). Pharmaceutical Care: The PCNE definition 2013. Int. J. Clin. Pharm..

[26] ASHP Medication Therapy and Patient Care, Organization and Delivery of Services–Statements. http://www.ashp.org/doclibrary/bestpractices/orgstpharmcare.aspx.

[27] Awad A., Al-Ebrahim S., Abahussain E. (2006). Pharmaceutical care services in hospitals of Kuwait. J. Pharm. Pharm. Sci..

[28] Miles A. (2016). The chronic illness problem. The person-centered solution. Eur. J. Pers. Cent. Healthc..

[29] Morgado M., Rolo S., Castelo-Branco M. (2011). Pharmacist intervention program to enhance hypertension control: A randomised controlled trial. Int. J. Clin. Pharm..

[30] Liekweg A., Westfeld M., Braun M., Zivanovic O., Schink T., Kuhn W., Jaehde U. (2012). Pharmaceutical care for patients with breast and ovarian cancer. Support. Care Cancer.

[31] Collins C., Limone B.L., Scholle J.M., Coleman C.I. (2011). Effect of pharmacist intervention on glycemic control in diabetes. Diabetes Res. Clin. Pract..

[32] Park H.Y., Seo S.A., Yoo H., Lee K. (2018). Medication adherence and beliefs about medication in elderly patients living alone with chronic diseases. Patient Prefer. Adherence.

[33] Piecuch A., Makarewicz-Wujec M., Kozłowska-Wojciechowska M. (2016). Improving the provision of OTC medication information in community pharmacies in Poland. Int. J. Clin. Pharm..

[34] Alder B., Abraham C., van Teijlingen E., Porter M. (2011). Psychology and Sociology Applied to Medicine E-Book.

[35] Jonikas M.A., Mandl K.D. (2011). Surveillance of medication use: Early identification of poor adherence. J. Am. Med. Inform. Assoc..

[36] Rutter P. (2015). Role of community pharmacists in patients’ self-care and self-medication. Integr. Pharm. Res. Pract..

[37] Burson R.C., Buttenheim A.M., Armstrong A., Feemster K.A. (2016). Community pharmacies as sites of adult vaccination: A systematic review. Hum. Vaccines Immunother..

